# Exploring How Evidence-Based Practice, Communication, and Clinical Simulation Outcomes Interact in Nursing Education: A Cross-Sectional Study

**DOI:** 10.3390/nursrep14010047

**Published:** 2024-03-13

**Authors:** José Jorge Carrasco-Guirao, César Leal-Costa, María de los Ángeles Castaño-Molina, Maria Belén Conesa-Ferrer, Alonso Molina-Rodríguez, José Luis Díaz-Agea, Maria Gracia Adánez-Martínez

**Affiliations:** 1 Faculty of Nursing, University of Murcia, Campus de Ciencias de la Salud, 30120 El Palmar-Murcia, Spain; josejorge.carrasco@um.es (J.J.C.-G.); angeles.castano@um.es (M.d.l.Á.C.-M.); mb.conesaferrer@um.es (M.B.C.-F.); alonso.molina@um.es (A.M.-R.); 2 Faculty of Medicine, University of Murcia, Campus de Ciencias de la Salud, 30120 El Palmar-Murcia, Spain; g.adanez@um.es

**Keywords:** nursing, communication skills, evidence-based practice, clinical simulation

## Abstract

(1) Background: Clinical simulation is an educational approach that aims to replicate real-life scenarios. Its primary goal is to help nursing students acquire the necessary knowledge and skills to perform effectively in clinical settings. This study focuses on the relationship between communication skills, evidence-based practice (EBP), and clinical simulation. We aimed to assess how communication skills and EBP competencies affect nursing students’ performance in simulated clinical scenarios. (2) Methods: We conducted an observational, cross-sectional study with 180 third-year nursing students at the University of Murcia. We used validated instruments to evaluate the students’ EBP competencies, communication skills, non-technical skills, and nursing interventions in simulated scenarios. (3) Results: The results showed that the students had varying competencies in EBP and communication skills. However, there was a positive and statistically significant correlation (*p* < 0.001) between these variables, non-technical skills, and the simulated clinical scenario nursing interventions. Our regression models revealed that communication skills and EBP competence significantly influenced the performance of the student nurses regarding their clinical and non-technical skills in the simulated scenarios. (4) Conclusions: Communication skills and EBP competencies predict performance in simulated scenarios for nursing students.

## 1. Introduction

The International Nursing Association of Clinical Simulation and Learning (INACSL) defines a simulation as “an educational strategy in which a particular set of conditions are created or replicated to resemble authentic situations that are possible in real life. Simulation can incorporate one or more modalities to promote, improve, or validate a participant’s performance” [1]. Clinical simulations enable nursing students to experience a representation of a real-life scenario, facilitating practice, learning, and the acquisition of necessary knowledge for proficient performance in diverse clinical scenarios that simulate real-world practice [2,3].

There are numerous simulation models. Zone-based simulation training (SimZones) [4] is structured into five levels, allowing the gradual training of skills and knowledge by increasing the fidelity and distraction levels to create more realistic environments. Zone 0 is for individual practices using virtual technology, while Zone 1 focuses on basic clinical skills. Zone 2 addresses more complex scenarios with clinical teams, emphasizing contextualized skills. Zone 3 involves native teams and system development, and Zone 4 focuses on post-patient care analysis. Starting from Zone 2, situations resembling daily practice are simulated, as was the case in the research presented in this article.

The learning process through clinical simulations is structured into various phases: pre-briefing, briefing, development of the simulated scenario, and debriefing [3]. It is noteworthy to emphasize the existence of specific standards for the planning and execution of clinical simulations in nursing education [5].

Clinical simulation allows individuals to experience clinical situations they may not have encountered during clinical practices, to make mistakes, and to subsequently enhance their performances by repeating them as often as necessary within a controlled environment without risk to the patient [6,7,8]. Using clinical simulations enhances the acquisition of clinical and non-technical skills, knowledge, handling of complex issues, self-confidence, communication skills, and clinical performance [9,10].

In health science disciplines, the emphasis is placed on students’ acquisition of specific skills that allow them to respond to current social and professional demands. Communication skills are the most sought after by university students in health sciences and are of significant importance, enhancing their interdisciplinary teamwork skills and interactions with health service users [11,12].

Nursing students must develop various communication skills, such as assertiveness, respect, active listening, and empathy. These skills enhance learning opportunities, promote patient safety, reduce errors in clinical settings, and provide patient-centered care [13,14,15].

Previous studies highlight the need for improvement in communication skills and indicate the challenges that nursing students face in understanding patients’ needs and emotions [16,17,18]. In the literature, we found references to the relationship between communication skills and the performance of nursing students in academic and clinical settings (simulated and real) [16,19,20,21].

Evidence-based practice (EBP) is grounded in problem-solving and gathering, analyzing, and applying research findings to enhance patient care and outcomes. Therefore, it is a fundamental cornerstone in nursing practice, integrating robust research, clinical experience, and patient preferences for informed decision-making [22,23]. EBP ensures quality, patient-centered care based on current research and knowledge, which is fundamental to nursing care delivery [24,25].

The relationship between clinical simulation and EBP in nursing education is significant. Simulation is a teaching method backed by evidence that is used to teach the best practices in nursing. This approach provides an opportunity to incorporate EBP into the learning scenario objectives [26]. Simulations can be valuable in teaching nursing students EBP competencies [27,28]. Although there is no direct scientific evidence, it is suggested that the EBP competencies of nursing students could enhance their performance in real and simulated clinical practice. This is because they can apply the most current knowledge and informed judgment, leading to better decision-making and a higher quality of care.

This study hypothesized that nursing students with practical communication skills and EBP competencies would perform better in simulated clinical scenarios. This study aimed to assess the relationship between communication skills and EBP competencies in nursing students and how they impact performance in simulated clinical scenarios.

## 2. Materials and Methods

### 2.1. Design

A cross-sectional, observational study was conducted on nursing students enrolled in the Nursing Degree program at the University of Murcia in Spain. This study adheres to the STROBE guidelines for reporting cross-sectional studies.

### 2.2. Sample and Setting

The target population consisted of third-year students in the bachelor’s degree in nursing program at the University of Murcia (Spain) enrolled in the subject Practicum 2 during the 2021/2022 academic year. A non-probabilistic sampling method was utilized because all the third-year students received HFS-based training as part of the curriculum.

The inclusion criteria were: (1) students in the third year of the Degree in Nursing, (2) students who attended all the clinical simulation sessions in zone 2, (3) those who adequately completed all the evaluation questionnaires, and (4) those who gave informed consent. The exclusion criteria were: (1) students had previously participated in any clinical simulation-based training in zone 2.

A bachelor’s degree in nursing in Spain takes four years and is divided into two four-month terms (15 weeks) per year. The first term runs from September to December, while the second runs from February to June. Every year, students enroll in courses for both semesters. During the first term of the third year, Practicum 2 takes place and lasts for 13 weeks. The student nurses in Practicum 2 receive clinical simulation-based training in zone 2 [4] and have not previously undertaken any clinical simulation-based training in zone 2.

All the students completed clinical scenarios in a simulated hospital with a high-fidelity patient simulator following INACSL standards [5]. Six scenarios were designed, four for internal medicine and two for surgery, following INACSL design recommendations [29]. The scenarios were created using the nursing language of the Nursing Intervention Classification (NIC) [30] (Table S1). The simulations were conducted by a facilitator with more than ten years of experience in conducting simulations, specifically in zone 2.

The clinical simulation-based training included a pre-briefing to establish a psychologically safe environment using group dynamics and best practice guidelines, followed by a scenario briefing that was delivered to the students [31,32]. Subsequently, the simulation of the clinical scenario was carried out in groups of 2–3 students, who were recorded and viewed in real-time by the rest of the students during the clinical simulation sessions. A structured debriefing was conducted following the GAS method (gather, analyze, and summarize) and guided by the INACSL guidelines [33].

### 2.3. Variables and Measurement Instruments

This study took into account the following variables:

1. Sociodemographic characteristics of the participants: age, sex.
2. Competence in evidence-based practice questionnaire (EBP-COQ) [34]. A Spanish instrument was developed to assess the level of self-perceived EBP competence in the nursing students. Before clinical simulation-based training, it evaluated the students’ knowledge (range 6–30), skills (range 6–30), and attitudes (range 13–65) toward EBP. The questionnaire demonstrated satisfactory psychometric properties, with a Cronbach’s alpha of 0.888 for the entire questionnaire. The factor solution accounted for 55.55% of the variance.
3. Communication skills scale (CSS) [35]. This is a validated questionnaire designed for health science students in Spain to evaluate communication skills among nursing students. The questionnaire includes 18 items divided into four categories: empathy (range 5–30), informative communication (range 6–36 points), respect (range 3–18), and assertiveness (range 4–24). The questionnaire shows good internal consistency, with Cronbach’s alpha scores of 0.77 for empathy, 0.78 for informative communication, 0.74 for respect, and 0.65 for assertiveness. A final model was tested with four oblique factors. The model fit indices were χ^2^ = 220.61 (df = 130; *p* < 0.001), CFI = 0.97, TLI = 0.98, and RMSEA = 0.05 (90% CI = 0.04–0.06). The questionnaire’s construct validity was confirmed by a correlation with burnout.
4. Non-technical Skills scale in medical and surgical hospital units for nursing students (NTS-Nursing) [36]: A validated version of this scale has been developed for Spanish nursing students to measure their non-technical skills in simulated clinical scenarios. The questionnaire includes ten items divided into three categories: teamwork (range 4–40), intervention management (range 4–40), and patient/family communication (range 2–20). The total score ranges from 10 to 100. The internal consistency (Cronbach’s α) for each dimension of the scale was 0.874 for teamwork, 0.861 for interventions management, and 0.967 for patient/family communication. The ICC showed a high agreement between the scores of the scale completed by the three evaluators: teamwork = 0.940 (*p* < 0.001); interventions management = 0.984 (*p* < 0.001); and patient/family communication = 0.982 (*p* < 0.001). The final model of 4 oblique factors with the 10 final items were tested. The fit indices of the CFA showed the following values: χ^2^ = 68.93 (df = 32; *p* < 0.001), CFI = 0.996, TLI = 0.995, and RMSEA= 0.05 (90% IC = 0.037–0.072). There is adequate evidence of its external validity, and all the dimensions demonstrated statistically significant correlations with the total in the NIC interventions.
5. To assess clinical skills in the simulated clinical scenarios, the nursing interventions (NICs) were used [30]. A checklist was created with five crucial activities of the NIC interventions to be evaluated in each simulated clinical scenario. The activities were selected through the consensus of two experts with more than ten years of experience in clinical simulation. Each activity performed by the students was scored with two points, allowing for a minimum score of zero and a maximum score of ten for each intervention.

### 2.4. Data Collection

All the data were collected between October and December of 2021. Before participating in the clinical simulation-based training, the participants were given an electronic questionnaire that included the communication skills scale (CSS), competence in evidence-based practice questionnaire (EBP-COQ), and socio-demographic variables. The University of Murcia Surveys tool was used to administer the questionnaire. An expert in clinical simulation evaluated the video recordings of the clinical scenarios based on the non-technical skills scale in medical and surgical hospital units for nursing students (NTS-Nursing) and the students’ clinical skills (NIC nursing interventions).

### 2.5. Ethical Considerations

This study was conducted in accordance with the ethical principles outlined in the Declaration of Helsinki [37]. The nursing students’ instructors were informed that their participation in the study was voluntary. The students were guaranteed that there would be no negative academic consequences should they refuse or withdraw from the study after agreeing to participate. To maintain confidentiality and anonymity, alphanumeric codes were assigned to the questionnaires, and no identifying information was collected from the participants. The anonymization process only applies to the collected data and not to the participants themselves. Informed consent was obtained from all the participants. In addition, the Ethics Committee of the University of Murcia approved the study (CEI) (code 3762).

### 2.6. Data Analysis

Descriptive statistics were used to summarize the characteristics of the sample and scores obtained based on the measurement instruments. The categorical variables were summarized based on frequency and percentage, and the quantitative variables were summarized using means and standard deviations (SDs).

Pearson’s r correlation coefficient was used to assess the relationship between the students’ performance in the simulated clinical scenarios (non-technical skills and nursing interventions), communication skills (informative communication, empathy, respect, and assertiveness), and EBP competence (knowledge, skills, and attitudes towards EBP).

A univariate and multivariate analysis (using forward stepwise selection) was conducted to investigate the relationship between the students’ communication skills, EBP competence, and performance in the simulated clinical settings based on their non-technical skills and nursing interventions.

A significance level of *p* ≤ 0.05 was used for the statistical analysis. SPSS (Statistical Package for the Social Sciences) v. 24.0 was used for the analysis.

## 3. Results

### 3.1. Description of the Participants

The final sample consisted of 180 3rd year nursing students. The mean age was 22.94 (SD = 7.61) years, and 82.8% of the students (*n* = 149) were female. Table 1 shows the main socio-demographic characteristics of the participants.

### 3.2. Results of the Tool Measurements

Table 1 presents the average scores for each dimension and the total scores of the instruments used. The results show that the nursing students received average scores for the skills and knowledge dimensions of the EBP-COQ. On the other hand, the mean scores for the attitudes dimension of the EBP-COQ, CSS dimensions, as well as the dimensions and the total scores of the NTS-Nursing and nursing interventions were rated at an average to high level.

### 3.3. Association between Clinical and Non-Technical Skills, Communication Skills, and Evidence-Based Practice Competence

According to the bivariate analysis, all the study variables had a positive correlation, including non-technical skills, nursing interventions, EBP competence, and communication skills. The coefficients were moderately high and statistically significant. This means that higher communication skills and EBP competence led to higher scores for the nursing students in non-technical skills and nursing interventions during the simulated clinical scenarios, as indicated in Table 2.

Table 3 displays the univariate and multivariate linear regression model with the dependent variables of non-technical skills and nursing interventions.

In the univariate linear regression analysis, all the independent variables, except for the attitude dimension of the EBP-COQ, showed a strong association with the dependent variables, with a probability of error of less than 0.001. The variance in communication skills explained between 31% and 61% of non-technical skills and between 28% and 54% of nursing interventions. Meanwhile, the variance in the knowledge and skills dimensions of EBP demonstrated a variation between 87% and 89% for non-technical skills and 72% and 79% for nursing interventions.

The multivariate linear regression statistical model was used to analyze the non-technical skills as the dependent variable. The results showed that the model could explain 90% of the variance, with an adjusted R^2^ value of 0.904. The t-test revealed a strong association between the variables included in the model, namely skills for EBP-COQ and respect for CSS, with an error probability of less than 0.001. The Durbin–Watson D-statistic suggested no autocorrelation in the residuals of the multiple linear regression model, with a value of D = 1.69. Furthermore, the tolerance and VIF values indicated no multicollinearity between the variables in this model.

The multivariate linear regression model used in this study, which employed nursing interventions as the dependent variable, could explain 81% of the variance (adjusted R^2^ = 0.814). The t-test revealed a significant association between the variables included in the model: the skill and knowledge dimensions of the EBP-COQ and the respect dimension of the CSS, with a probability of error of less than 0.001. The Durbin–Watson D-statistic test indicated the absence of autocorrelation in the residuals of the multiple linear regression model (D = 1.43). Additionally, the tolerance and VIF values supported the non-existence of multicollinearity among the predictor variables.

## 4. Discussion

This study examined how nursing students’ perceived communication skills and evidence-based practice (EBP) competencies impact their performance in simulated clinical scenarios. This study found that nursing students’ self-assessed communication skills and EBP competencies were significantly associated with their performance in the simulated clinical scenarios, indicating that these factors can predict their performance.

The participants’ positive self-perception of communication skills is reflected in their medium-high scores on the CSS. The students scored the highest in the “empathy” and “respect” categories, which is consistent with previous research [21,38,39,40].

It was found that there was a strong connection between the students’ communication skills and their non-technical skills and nursing interventions during the simulated clinical scenarios. The students with high levels of empathy, informative communication, respect, and assertiveness performed exceptionally well in these scenarios. These results were similar to another study [21], where a positive and statistically significant relationship was found between communication skills and the performance of nursing students in simulated clinical practice. However, the correlation coefficients we obtained in our study were higher than those in this previous study.

Several studies have shown that practical communication skills are essential for healthcare professionals, including nursing students, and can improve patient outcomes [22,41,42,43]. Our study explored the correlation between nursing students’ communication skills and their non-technical skills (such as teamwork and management of nursing interventions) and nursing interventions during simulated clinical scenarios. The findings were confirmed based on the univariate linear regression analysis. In this analysis, all the dimensions of CSS were significantly associated with the nursing students’ non-technical skills and nursing interventions, with a *p*-value of less than 0.001. The CSS dimensions accounted for variances between 31% and 61% for non-technical skills and between 28% and 54% for nursing interventions. Other studies have indicated that nurses with more empathy and emotional intelligence tend to have a more positive attitude towards communication [12]. Similarly, nursing students with better communication skills tend to perform better in simulated clinical practice scenarios [21].

The study participants showed average scores on the knowledge and skill dimensions of the EBP-COQ, with the highest scores attained for the attitude dimension. Similar results have been documented in previous research [44]. It is possible to explain the higher score in the attitude dimension among the nursing students by the presence of pre-existing positive attitudes towards using EBP. Attitudes toward EBP are influenced by various factors, including prior experience with nursing research [45]. Although it is possible that the students lacked research experience, they likely already recognized EBP as the optimal tool for effective care planning and implementation.

In addition, a positive, statistically significant correlation was found between the EBP-COQ skill and knowledge dimensions and the students’ performance in the non-technical skills and nursing interventions in the simulated clinical scenarios. The students with high levels of EBP skills and knowledge performed better in the simulated clinical scenarios. However, more scientifically rigorous studies are crucial to assess how these attributes, particularly knowledge and skills, contribute to a high performance in simulated clinical scenarios.

These data were corroborated based on the univariate linear regression analysis, where all the EBP-COQ skill and knowledge dimensions were significantly (*p* < 0.001) associated with the non-technical skills and nursing interventions presented by the nursing students. Several studies [22,24] have demonstrated that evidence-based practice (EBP) is essential for delivering care scientifically and ethically across diverse nursing practice settings. In our study, we investigated how the informed use of evidence-based care can enhance the performance of nursing students in simulated clinical scenarios.

The results of a multivariate linear regression analysis indicated that the two variables that most accurately explained the students’ non-technical skills were the respect dimension of the CSS and the skills dimension of the EBP-COQ. Other studies have used multivariate models to predict nurse communication attitudes [12,46]. These studies highlighted the importance of empathy and emotional intelligence as predictor variables, but the models’ variance was lower than in our study. A study conducted by Sánchez-Expósito et al. [21] presented a multivariate model in which several socio-emotional variables, including the communication skills of nursing students, accounted for 50% of the variance in the overall performance of the nursing students during simulated clinical practice scenarios.

There is a gap in the literature regarding the use of EBP competence to predict student performance in clinical simulations. However, previous research has shown that clinical simulation-based learning can enhance skills and knowledge in EBP. This is because students actively address the challenges of finding the best evidence for the simulation. They must formulate research questions and critically appraise identified articles [28]. From the initial phase preceding the simulation (pre-briefing), students are forced to search for evidence relevant to the scenarios and learning objectives. As a result, in our study, we found that the skill and knowledge dimensions of the EBP-COQ were positively correlated with the students’ performance in the simulated clinical scenarios.

The study presented here has some limitations that need to be considered. One such limitation concerns the sampling procedures and the geographical restriction of the participants. The sampling procedures were non-probabilistic, meaning the study’s results may not be generalizable to other populations. Additionally, the study focused only on students from a single university, which further limits the applicability of the findings. Future research may address this issue by implementing stratified probability sampling with geographic allocations and a multi-center approach to generate a more representative sample.

Another limitation is related to the use of self-reports to collect data. While self-reports are a standard tool in research, they can introduce a social desirability bias. To control for this, non-technical skills and nursing interventions were assessed by trained teachers who performed external objective measures. Overall, it is essential to be aware of these limitations in future research and consider them when interpreting this study’s results.

### Implications for Nursing Education

Effective communication skills and evidence-based practice (EBP) competencies are integral predictors of nursing students’ performance in simulated scenarios. Proficiency in communication ensures clear patient interaction, interprofessional collaboration, and accurate information exchange, which is vital for delivering safe and high-quality care. Additionally, EBP competencies equip students to critically evaluate evidence, make informed clinical decisions, and adapt to dynamic healthcare environments. Recognizing the significance of these skills in simulated scenarios underscores their pivotal role in nursing education, emphasizing the need for curricular integration and ongoing development to prepare students for real-world practice challenges.

## 5. Conclusions

The students received an average score based on their knowledge and skills related to EBP, but they demonstrated good levels of empathy, respect, informative communication, assertiveness, and attitude towards EBP.

This study found that the nursing students’ communication skills and EBP competence significantly predicted their performance in non-technical skills (teamwork, managing nursing interventions, and communicating with patients and their families) and nursing interventions (NIC).

## Figures and Tables

**Table 1:** Descriptive statistics of the characteristics of the sample.

Categorical Variables	*n*	%
Gender		
Male	149	82.8
Female	31	17.2
Quantitative Variables	*M*	*SD*
Age (years)	22.94	7.61
Skills (EBP-COQ) (range 6–30)	17.16	4.52
Knowledge (EBP-COQ) (range 6–30)	15.59	4.23
Attitudes (EBP-COQ) (range 13–65)	57.16	4.98
Informative communication (CSS) (range 6–36 points)	26.71	2.68
Empathy (CSS) (range 5–30)	24.72	3.31
Respect (CSS) (range 3–18)	15.16	2.08
Assertiveness (CSS) (range 4–24)	16.00	1.73
Teamwork (NTS-Nursing) (range 4–40)	31.02	2.61
Intervention management (NTS-Nursing) (range 4–40)	30.41	2.81
Patient/family communication (NTS-Nursing) (range 2–20)	14.99	1.58
Total non-technical skills (NTS-Nursing) (range 10–100)	76.43	6.42
Total nursing interventions classification (NIC) (range 1–10)	8.73	0.98

**Table 2:** Bivariate correlations between the dimensions and totals of the NTS-Nursing scale and the total score in the NIC nursing interventions based on the dimensions of the communication skills scale and evidence-based practice competence.

	IC	Emp	Resp	Assert	Skills—EBP	Knowledge—EBP	Attitudes—EBP
TW	0.641 **	0.709 **	0.664 **	0.512 **	0.907 **	0.890 **	0.096
IM	0.591 **	0.631 **	0.624 **	0.420 **	0.871 **	0.866 **	0.030
Com	0.810 **	0.876 **	0.826 **	0.678 **	0.799 **	0.776 **	0.117
NTS Total	0.718 **	0.780 **	0.746 **	0.559 **	0.947 **	0.932 **	0.081
Total NIC	0.708 **	0.748 **	0.738 **	0.528 **	0.935 **	0.913 **	0.079

** The correlation is significant at the 0.01 level (bilateral). NTS Total: Total score on the non-technical skills scale. NTS dimensions: TW: teamwork; IM: management of the nursing interventions; Com: patient/family communication. IC: informational communication; Emp: empathy, Resp: respect; Assert: assertiveness. Total NIC: total score based on the NIC nursing interventions. EBP: evidence-based practice.

**Table 3:** Univariate and multivariate linear regression models.

Dependent Variables	Non-Technical Skills							Nursing Interventions						
	Univariate				Multivariate			Univariate				Multivariate		
Independent Variables	*β*	*t*	*p*-Value	Adjusted R^2^	*β*	*t*	*p*-Value	*β*	*t*	*p*-Value	Adjusted R^2^	*β*	*t*	*p*-Value
Skills (EBP-COQ)	0.947	39.20	<0.001	0.89	0.850	25.43	<0.001	0.887	25.65	<0.001	0.79	1.228	7.12	<0.001
Knowledge (EBP-COQ)	0.932	34.24	<0.001	0.87				0.850	21.51	<0.001	0.72	0.492	2.95	0.004
Attitudes (EBP-COQ)	0.080	1.08	0.28	0.001				0.061	0.82	0.42	0.002			
Informative communication (CSS)	0.718	13.77	<0.001	0.61				0.710	13.38	<0.001	0.50			
Empathy (CSS)	0.780	16.63	<0.001	0.51				0.734	14.41	<0.001	0.54			
Respect (CSS)	0.746	14.96	<0.001	0.55	0.134	3.99	<0.001	0.738	14.61	<0.001	0.54	0.196	4.21	<0.001
Assertiveness (CSS)	0.559	8.98	<0.001	0.31				0.531	8.36	<0.001	0.28			
					R = 0.951; adjusted R^2^ = 0.904; F = 841.08; *p* < 0.001; Durbin–Watson’s D statistic = 1.69							R = 0.904; adjusted R^2^ = 0.814; F = 261.28; *p* < 0.001; Durbin–Watson’s D statistic = 1.43		

*β* = Standardized Coefficients.

## Data Availability

The data are available upon email request sent to the corresponding authors.

## Supplementary Materials

The following supporting information can be downloaded at: https://www.mdpi.com/article/10.3390/nursrep14010047/s1, Table S1: Scenarios in which students learned through high-fidelity simulation.

## Public Involvement Statement

There was no public involvement in any aspect of this research.

## Guidelines and Standards Statement

This manuscript was drafted based on the Strengthening the Reporting of Observational Studies in Epidemiology (STROBE) guidelines for cross-sectional studies.
