# Supplementary material for: Exploring How Evidence-Based Practice, Communication, and Clinical Simulation Outcomes Interact in Nursing Education: A Cross-Sectional Study
Source: Nurs Rep. 2024 Mar 13;14(1):616–26. doi: 10.3390/nursrep14010047 (PMC10975257; doi:10.3390/nursrep14010047)
Supplement: Supplementary file 1 [file nursrep-14-00047-s001.zip › nursrep-2847549-supplementary.pdf]

Supplementary Material

Table S1. Scenarios in which students learned through high-fidelity simulation.

Simulated Clinical Scenario	NIC Interventions
A patient with dyspnea, increased expectoration, and a 38.5 °C fever was admitted from the emergency department. The patient has difficulty breathing. Diagnosis: pneumonia.	- Vital signs monitoring (6680)<br>- Medication administration (2300)<br>- Oxygen therapy (3320)<br>- Phlebotomy: arterial blood sample (4232)
The patient has been admitted to the Cardiology department to manage non-ST elevation acute coronary syndrome (NSTEACS). Currently, the patient is in the ward and is waiting for a coronary angiography procedure. The patient's family has reported that the patient experiences chest pain and nausea while at rest.	- Cardiac care: acute (4044)<br>- Vital signs monitoring (6680)<br>- Phlebotomy: venous blood sample (4238)
The patient was admitted to the cardiology unit after experiencing acute coronary syndrome without ST elevation. It is worth noting that he had been hospitalized in the ICU ten days prior, during which time he underwent therapeutic catheterization and had two stents placed in his right coronary artery. The patient's wife, who is present with him, has expressed concerns about her husband's unresponsiveness. He is expected to be discharged from the hospital tomorrow.	- Resuscitation (6320)<br>- Defibrillator management: external (4095)<br>- Medication administration: intravenous (IV) (2314)
The patient was admitted to the internal medicine unit following a decompensation of Type I diabetes caused by endocrinological factors. Five days ago, the patient experienced diabetic ketoacidosis, which was treated in the intensive care unit (ICU) where he was initially admitted. The patient, who also has hypertension, is accompanied by his daughter who reports that he is experiencing symptoms of hyperglycemia.	- Hyperglycemia management (2120)<br>- Liquids/electrolytes management (2080)<br>- Medication administration: intravenous (IV) (2314)
A patient has been diagnosed with acute pancreatitis along with choledocholithiasis. After undergoing cholecystectomy surgery, the patient now has a left Penrose drain placed in the pouch of Douglas and a Jackson–Pratt drain in the pancreatic region. The patient is experiencing intense pain and nausea.	- Nausea management (1450)<br>- Pain management (1400)<br>- Tube care (1870)
The patient is scheduled as the third case on the traumatology surgery list to receive a prosthetic hip due to an accidental fall. She is currently feeling discouraged, insecure, fearful, and stressed, especially about her hygiene and whether she will be able to walk again after the surgery. Before entering the operating room, the patient needs to receive concentrated red blood cells as per the results of emergency analytical tests, which showed low hematocrit levels and red blood cell values.	- Teaching: preoperative (5610)<br>- Surgical preparation (2930)<br>- Blood products administration (4030)
